# Whole-Genome Sequence of Acinetobacter baumannii HUMV-3743, Isolated from a Human Wound Exudate

**DOI:** 10.1128/MRA.01640-18

**Published:** 2019-05-09

**Authors:** Itziar Chapartegui-González, María Lázaro-Díez, Santiago Redondo-Salvo, Javier Gonzalo Ocejo-Vinyals, Jesús Navas-Méndez, José Ramos-Vivas

**Affiliations:** a Instituto de Investigación Sanitaria Valdecilla (IDIVAL), Santander, Spain; b Servicio de Microbiología, Hospital Universitario Marqués de Valdecilla, Santander, Spain; c Red Española de Investigación en Patología Infecciosa (REIPI), Instituto de Salud Carlos III, Madrid, Spain; d Instituto de Biomedicina y Biotecnología de Cantabria (IBBTEC), Universidad de Cantabria, Santander, Spain; e Servicio de Inmunología, Hospital Universitario Marqués de Valdecilla, Santander, Spain; f Departamento de Biología Molecular, Universidad de Cantabria, Santander, Spain; University of Maryland School of Medicine

## Abstract

Acinetobacter baumannii strain HUMV-3743 was obtained from wound exudate from an adult patient. Here, we report its complete genome sequence using Illumina-based sequence analysis, which revealed a genome of 4 Mb, which includes 2 predicted plasmids of 78.9 and 107 kb. A total of 3,881 protein-coding genes are predicted from this assembly.

## Abstract (precis)

Acinetobacter baumannii strain HUMV-3743 was obtained from wound exudate from an adult patient. Here, we report its complete genome sequence using Illumina-based sequence analysis, which revealed a genome of 4 Mb, which includes 2 predicted plasmids of 78.9 and 107 kb.

## ANNOUNCEMENT

Acinetobacter species are inherently resistant to several antibiotics or capable of readily acquiring resistance. Clinical isolates are able to rapidly spread among patients and survive in the hospital environment (1, 2). Acinetobacter baumannii has been extensively studied because it has been associated with a high mortality rate and has emerged as one of the most problematic nosocomial pathogens (3). Like other Gram-negative bacilli, A. baumannii has a great ability to acquire a multidrug-resistant (MDR) phenotype. For this reason, in 2017, the World Health Organization (WHO) published a list of MDR and extensively drug-resistant (XDR) bacteria for which new antibiotics are urgently needed, with carbapenem-resistant A. baumannii (CRAB) being the first critical priority (4).

The strain used in this study (HUMV-3743) was isolated in blood agar from the wound exudate of an adult at the Hospital Universitario Marqués de Valdecilla (HUMV) in Santander, Spain. The strain was routinely cultured in blood agar, Luria agar, or Luria broth at 37°C and frozen at −80°C with 20% glycerol.

The total genomic sample of A. baumannii strain HUMV-3743 was extracted and purified using the GeneJET genomic DNA (gDNA) isolation kit (Thermo Scientific) after growth on Luria broth for 24 h at 37°C. The gDNA was submitted to Fundació per al Foment de la Investigació Sanitària i Biomèdica (Fisabio; Spain) for Illumina (MiSeq 2 × 300-bp) sequencing. DNA libraries were generated following the Nextera XT Illumina protocol (Nextera XT library prep kit [catalog number FC-131-1024]). We used 0.2 ng/µl purified gDNA to initiate the protocol. The mutiplexing step was performed using the Nextera XT index kit (catalog number FC-131-1096). The libraries were sequenced using a 2 × 300-bp paired-end run (MiSeq v3 reagent kit [catalog number MS-102-3003] on a MiSeq sequencer, according to the manufacturer’s instructions [Illumina]).

The whole-genome sequence was assembled using Unicycler v0.3.0.b (5), resulting in 176 contigs, with a total genome size of 4,066,761 bp, a GC content of 39.0%, an *N*_50_ value of 111,502 bp, and an *L*_50_ value of 12. Quality control was performed with prinseq-lite (6) with the following parameters: min. length, 50; trim_qual_right, 30; trim_qual_type, mean; and trim_qual_window, 20. Total reads (2,152,955) R1 and R2 of the Illumina sequencing run were joined using FLASH (7). Two probable plasmids of approximately 107 kb and 78.9 kb were predicted from this genome with the PLACNETw server (8).

Annotation of the genome sequence using the Rapid Annotations using Subsystems Technology (RAST) server (9) identified 3,881 coding sequences and 66 RNAs (63 tRNA genes and 1 mRNA genes) with the ARAGORN software (10) and 2 rRNAs with the RNAmmer software (11). Using RAST, 456 subsystems were predicted, including those involved in carbohydrate metabolism (n = 314), protein metabolism (n = 225), synthesis of amino acids and derivatives (445), cell wall and capsule synthesis (n = 123), RNA metabolism (n = 128), DNA metabolism (256 in cofactors, vitamins, and prosthetic groups and 99 in nucleoside and nucleotide synthesis), fatty acid and lipid synthesis (n = 178), virulence (n = 71), membrane transport (n = 108), phosphorus metabolism (n = 40), regulation and cell signaling (n = 85), secondary metabolism (n = 5), phages, prophages, transposable elements, and plasmids (n = 40), stress response (n = 117), and dormancy and sporulation (n = 2).

Gene clusters related to siderophores (actinoferrin), aryl polyene, and others (12) were detected using antiSMASH (bacterial version). A preliminary analysis using Prokka 1.13 (13) and the ABRicate 0.8 software (https://www.github.com/tseemann/abricate), as well as CARD (14), identified the following antimicrobial resistance genes: *ant(3″)-IIa, aph(6)-Id*, *aph(3′)-VIa*, *aph(3″)-Ib* (also named *strA*), and *aac(3)-IIa*, related to aminoglycoside resistance; *ampC*, *bla*_OXA-109_, and *bla*_ADC-25_, related to β-lactam resistance; *tetA*, related to tetracycline resistance; and *catB4*, related to chloramphenicol resistance. This analysis also revealed a broad repertoire of efflux pump genes (*adeABC, adeFGH, adeIJK, abeS,* and *abeM,* among others), most of which are associated with antimicrobial resistance as well.

### Data availability.

This whole-genome shotgun project has been deposited at DDBJ/ENA/GenBank under the accession number RRCI00000000 and BioProject accession number PRJNA507520. The version described in this paper is version RRCI01000000.
